# Serum alkaline phosphatase levels and the risk of new-onset diabetes in hypertensive adults

**DOI:** 10.1186/s12933-020-01161-x

**Published:** 2020-10-24

**Authors:** Yuanyuan Zhang, Chun Zhou, Jianping Li, Yan Zhang, Di Xie, Min Liang, Binyan Wang, Yun Song, Xiaobin Wang, Yong Huo, Fan Fan Hou, Xiping Xu, Xianhui Qin

**Affiliations:** 1 Division of Nephrology, Nanfang Hospital, Southern Medical UniversityNational Clinical Research Center for Kidney DiseaseState Key Laboratory of Organ Failure ResearchGuangdong Provincial Institute of Nephrology, Guangdong Provincial Key Laboratory of Renal Failure Research, Guangzhou Regenerative Medicine and Health Guangdong Laboratory, Guangzhou, 510515 China; 2 Department of Cardiology, Peking University First Hospital, Beijing, 100034 China; 3 Institute of Biomedicine, Anhui Medical University, Hefei, 230032 China; 4 Beijing Advanced Innovation Center for Food Nutrition and Human Health, College of Food Science and Nutritional Engineering, China Agricultural University, Beijing, 100083 China; 5 Department of Population, Family and Reproductive Health, Johns Hopkins University Bloomberg School of Public Health, 615 N. Wolfe Street, E4132, Baltimore, MD 21205-2179 USA

**Keywords:** Alkaline phosphatase, New-onset diabetes, New-onset impaired fasting glucose, Total homocysteine, Hypertension

## Abstract

**Background:**

The association between alkaline phosphatase (ALP) and incident diabetes remains uncertain. Our study aimed to investigate the prospective relation of serum ALP with the risk of new-onset diabetes, and explore possible effect modifiers, in hypertensive adults.

**Methods:**

A total 14,393 hypertensive patients with available ALP measurements and without diabetes and liver disease at baseline were included from the China Stroke Primary Prevention Trial (CSPPT). The primary outcome was new-onset diabetes, defined as physician-diagnosed diabetes or use of glucose-lowering drugs during follow-up, or fasting glucose ≥ 7.0 mmol/L at the exit visit. The secondary study outcome was new-onset impaired fasting glucose (IFG), defined as FG < 6.1 mmol/L at baseline and ≥ 6.1 but < 7.0 mmol/L at the exit visit.

**Results:**

Over a median of 4.5 years follow-up, 1549 (10.8%) participants developed diabetes. Overall, there was a positive relation of serum ALP and the risk of new-onset diabetes (per SD increment, adjusted OR, 1.07; 95% CI: 1.01, 1.14) and new-onset IFG (per SD increment, adjusted OR, 1.07; 95% CI: 1.02, 1.14). Moreover, a stronger positive association between baseline ALP (per SD increment) with new-onset diabetes was found in participants with total homocysteine (tHcy) < 10 μmol/L (adjusted OR, 1.24; 95% CI: 1.10, 1.40 vs. ≥ 10 μmol/L: adjusted OR, 1.03; 95% CI: 0.96, 1.10; *P*-interaction = 0.007) or FG ≥ 5.9 mmol/L (adjusted OR, 1.16; 95% CI: 1.07, 1.27 vs. < 5.9 mmol/L: adjusted OR, 1.00; 95% CI: 0.93, 1.08; *P*-interaction = 0.009)

**Conclusions:**

In this non-diabetic, hypertensive population, higher serum ALP was significantly associated with the increased risk of new-onset diabetes, especially in those with lower tHcy or higher FG levels.

*Clinical Trial Registration-URL* Trial registration: NCT00794885 (clinicaltrials.gov). Retrospectively registered November 20, 2008.

## Background

Diabetes mellitus has been a public issue with increasing prevalence worldwide [1, 2]. The global diabetes prevalence in 2019 is estimated to be 9.3% (463 million people), projected to reach 10.2% (578 million) by 2030 and 10.9% (700 million) by 2045 [1]. Diabetes result in many complications, including cardiovascular disease (CVD) and chronic kidney diseases (CKD), amputation and vision problems [3, 4]. The identification of more modifiable risk factors may possibly reduce the huge burden of diabetes and its associated complications by leading to early detection and prevention.

Prior studies have reported that liver function may be associated with diabetes [5, 6]. Alkaline phosphatase (ALP) is a generally accepted clinical marker of hepatic or bone disease [7]. It had been showed that elevated ALP acted as a prognostic indicator of decreased survival in diabetic patients with acute myocardial infarction (MI), possibly in association to decreased renal function in male patients [8]. At the same time, the combined effect of vascular calcification (VC) and higher ALP was associated with a greater risk of cardiovascular events and death, and high serum ALP increased the risk associated with VC in end-stage kidney disease patients starting dialysis [9]. Moreover, in a nest case–control study, ALP in type 2 diabetes seemed to be associated with CVD risk and stroke incidence in men, but with borderline significance [10]. However, only a few previous prospective studies [11–14] have been carried out to evaluate the relation of ALP and incident diabetes, and reported inconsistent results. In addition, although hypertension is one of the important risk factors for diabetes [3, 15, 16], few related studies has been conducted in hypertensive patients. More importantly, potential modifiers on the association between ALP and incident diabetes have not been comprehensively examined in previous studies.

This study was motivated by the limited and inconclusive evidence regarding the ALP levels and incident diabetes, and a special opportunity to address this question in a large, randomized controlled trial with regular antihypertensive treatments, BP measurements and diabetes status reports. Specifically, using data from China Stroke Primary Prevention Trial (CSPPT) [17], we aimed to investigate the prospective association between serum ALP and new-onset diabetes among hypertensive adults, and to examine possible modifiers on the association.

## Methods

### Study design and participants

The study design, methods and major results of the CSPPT (ClinicalTrials.gov identifier NCT00794885) have been reported elsewhere in detail [17–22]. Briefly, the CSPPT was a multi-community, randomized, double-blind, controlled trial conducted from May 19, 2008 to August 24, 2013 in 32 communities in Anhui and Jiangsu provinces in China. Eligible participants were men and women aged 45–75 years who had hypertension, defined as seated, resting systolic blood pressure (SBP) ≥ 140 mmHg or diastolic blood pressure (DBP) ≥ 90 mmHg at both the screening and recruitment visit, or who were on anti-hypertensive medication. The major exclusion criteria included history of physician-diagnosed stroke, myocardial infarction (MI), heart failure, post-coronary revascularization, and/or congenital heart disease, and/or current supplementation by folic acid, vitamin B12 or vitamin B6.

**Fig. 1:**
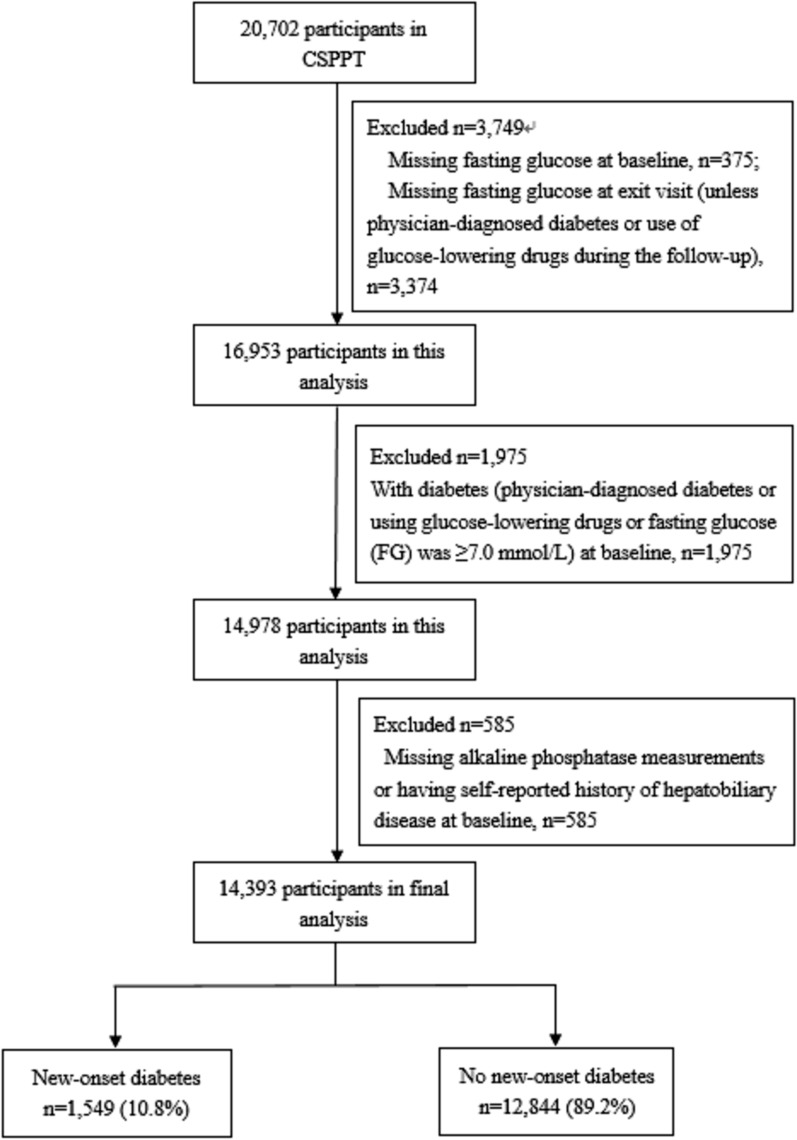
Flow chart of the participants

In the CSPPT, a total of 20,702 eligible participants were enrolled. Our current study is a post-hoc analysis of the CSPPT, including a total of 14,393 participants with complete major data and who were free of diabetes [physician-diagnosed diabetes or using glucose-lowering drugs or fasting glucose (FG) was < 7.0 mmol/L (126 mg/dL)], as well as without liver disease (self-reported chronic hepatitis, hepatic adipose infiltration, or cirrhosis) at baseline (Fig. 1).

### Intervention and follow-up

Eligible participants were randomized to receive a daily oral dose of 1 tablet containing 10 mg enalapril and 0.8 mg folic acid (single pill combination, the enalapril-folic acid group) or one tablet containing 10 mg enalapril only (the enalapril-only group).

Participants were scheduled for follow-up every 3 months. At each follow-up visit, BP was measured; study drug compliance, concomitant medication use, adverse events and possible endpoint events were documented by trained research staff and physicians.

### Laboratory assessment

Serum fasting ALP, gamma glutamyl transpeptidase (GGT), alanine aminotransferase (ALT), aspartate aminotransferase (AST), total homocysteine (tHcy), creatinine, lipids and fasting glucose (FG) were measured with the use of automatic clinical analyzers (Beckman Coulter) at the core laboratory of the National Clinical Research Center for Kidney Disease, Nanfang Hospital, Guangzhou, China. Serum folate at baseline were measured by a commercial lab using a chemiluminescent immunoassay (New Industrial, Shenzhen, China). Estimated glomerular filtration rate (eGFR) was calculated using the Chronic Kidney Disease Epidemiology Collaboration (CKD-EPI) equation [23].

### Study outcomes

The primary study outcome was new-onset diabetes, defined as physician-diagnosed diabetes, or use of glucose-lowering drugs during follow-up, or new onset FG ≥ 7.0 mmol/L (126 mg/dL) at the exit visit. In China, the clinical diagnosis and treatment of diabetes used the same criteria according to the China guideline for diabetes [24, 25].

The secondary study outcome was new-onset impaired fasting glucose (IFG), defined as FG < 6.1 mmol/L (110 mg/dL) at baseline and ≥ 6.1 mmol/L but < 7.0 mmol/L at the exit visit. The analysis of new-onset IFG included subjects whose FG < 6.1 mmol/L and without new-onset diabetes during the follow-up.

### Statistical analysis

Baseline characteristics are presented as means ± standard deviations (SDs) or medians [interquartile range (IQR)] for continuous variables and proportions for categorical variables. Statistical significance of differences in baseline characteristics was assessed in accordance with baseline serum ALP quartiles (< 79, 79 to < 96, 96 to < 116, and ≥ 116 IU/L) using ANOVA tests, signed rank tests or chi-square tests, accordingly.

We first explored the association between serum ALP and new-onset diabetes using thin plate regression splines in generalized additive models implemented by the R package *mgcv*. Then multivariable logistic regression models [odds ratio (OR) and 95% confidence interval (CI)] were used to evaluate relation of serum ALP with new-onset diabetes and new-onset IFG, without and with adjustment for age, sex, study center, treatment group, body mass index (BMI), smoking, alcohol drinking, family history of diabetes, SBP, FG, total cholesterol (TC), triglycerides (TG), eGFR, folate, tHcy and the use of antihypertensive drugs at baseline, as well as time-averaged SBP during the treatment period. As additional exploratory analyses, possible modifications on the association between serum ALP and new-onset diabetes were also evaluated by stratified analyses and interaction testing.

A two-tailed *P* < 0.05 was considered statistically significant in all analyses. R software, version 3.6.3 (https://www.R-project.org/) was used to perform all statistical analyses.

## Results

### Study participants and baseline characteristics

In this study, a total of 14,393 participants with complete major data and without diabetes and liver disease at baseline, were included in the final analyses (Fig. 1).

Baseline characteristics of participants by baseline ALP quartiles are presented in Table 1. The mean and median serum ALP levels were 100 IU/L (SD, 30.5) and 96 IU/L, respectively. Participants with higher ALP levels were older and more likely to be female; had higher SBP, TG, high-density lipoprotein (HDL) cholesterol, FG, folate levels at baseline and time-averaged on-treatment SBP during the treatment period; lower BMI, DBP, TC, tHcy levels at baseline and time-averaged on-treatment DBP during the treatment period; and lower frequency in use of antihypertensive drugs and antiplatelet drugs at baseline, as wells as lower frequency of current smoking, alcohol drinking and family history of diabetes (Table 1).

**Table 1:** Characteristics of the study participants by baseline serum alkaline phosphatase (ALP) quartiles

Variables^*^	Serum ALP, IU/L				*P* value^†^
	Q1 (< 79)	Q2 (79- < 96)	Q3 (96- < 116)	Q4 (≥ 116)	
N	3486	3623	3577	3707	
Age, yr	58.3 ± 8.1	59.7 ± 7.5	60.7 ± 7.1	61.3 ± 6.6	< 0.001
Male, no. (%)	1773 (50.9)	1609 (44.4)	1377 (38.5)	1059 (28.6)	< 0.001
Body mass index, kg/m^2^	25.4 ± 3.6	25.1 ± 3.6	24.8 ± 3.6	24.4 ± 3.7	< 0.001
Current smoking, no. (%)	963 (27.6)	938 (25.9)	810 (22.6)	664 (17.9)	< 0.001
Current drinking, no. (%)	1143 (32.8)	949 (26.2)	778 (21.8)	567 (15.3)	< 0.001
Family history of diabetes, no. (%)	151 (4.3)	145 (4.0)	125 (3.5)	116 (3.1)	0.036
Enalapril group, no. (%)	1727 (49.5)	1838 (50.7)	1800 (50.3)	1850 (49.9)	0.769
BP, mmHg					
SBP at baseline	165.6 ± 20.5	167.2 ± 20.4	167.5 ± 20.5	168.2 ± 20.3	< 0.001
DBP at baseline	95.4 ± 11.9	94.5 ± 11.9	94.3 ± 11.8	93.3 ± 11.7	< 0.001
Time-averaged SBP	138.4 ± 10.6	139.1 ± 10.3	138.8 ± 10.6	139 ± 10.6	0.022
Time-averaged DBP	84.1 ± 7.2	83.3 ± 7.2	82.6 ± 7.0	81.7 ± 7.3	< 0.001
Laboratory results, mmol/L					
Total cholesterol	5.6 ± 1.1	5.6 ± 1.1	5.5 ± 1.1	5.3 ± 1.1	< 0.001
Triglycerides	1.6 ± 1.9	1.6 ± 0.9	1.6 ± 0.9	1.7 ± 0.9	< 0.001
HDL cholesterol	1.3 ± 0.4	1.3 ± 0.4	1.4 ± 0.4	1.4 ± 0.4	< 0.001
Fasting glucose	5.5 ± 0.7	5.4 ± 0.7	5.4 ± 0.7	5.3 ± 0.7	< 0.001
eGFR, mL/min1.73/m^2^	93.7 ± 13.1	93.8 ± 12.8	93.3 ± 12.1	93.6 ± 12.2	0.455
Folate, ng/mL	7.8 ± 3.3	8.2 ± 3.8	8.5 ± 3.8	9.3 ± 4.4	< 0.001
Total homocysteine, μmol/L	14.9 ± 9.9	14.5 ± 8.8	14.5 ± 8.0	14.1 ± 6.9	< 0.001
Alkaline phosphatase, IU/L	66.1 ± 10.4	87.1 ± 4.8	104.9 ± 5.8	140.0 ± 24.1	< 0.001
Aspartate transaminase, IU/L	21.4(18.0,26.0)	22.7(19.2,27.8)	24.0(20.1,29.5)	26.7(21.9,33.1)	< 0.001
Alanine transaminase, IU/L	11.0(8.0,14.1)	12.0(9.0,16.0)	12.8(10.0,17.0)	14.0(11.0,19.0)	< 0.001
Gamma glutamyl transpeptidase, IU/L	19.1(14.3,27.6)	19.3(14.6,27.5)	19.3(14.6,28.1)	19.9(14.9,28.7)	0.005
Medication use, no. (%)					
Antihypertensive drugs	1794 (51.5)	1708 (47.1)	1621 (45.3)	1531 (41.3)	< 0.001
Lipid lowering drugs	37 (1.1)	26 (0.7)	19 (0.5)	25 (0.7)	0.065
Antiplatelet drugs	161 (4.6)	109 (3.0)	102 (2.9)	71 (1.9)	< 0.001

*ALP* serum alkaline phosphatase, *DBP* diastolic blood pressure, *eGFR* estimated glomerular filtration rate, *HDL* high-density lipoprotein, *SBP* systolic blood pressure

^*^Continuous variables are presented as Mean ± SD or IQR (25, 75th), categorical variables are presented as n (%)

^†^Difference between any 2 groups

In addition, during the treatment period, participants with higher ALP levels had higher frequency in use of calcium channel blockers; lower frequency in use of diuretics and antiplatelet drugs. (Table 2).

**Table 2:** Concomitant medication usage during the treatment period by baseline serum alkaline phosphatase quartiles

Variables^*^	Baseline serum alkaline phosphatase quartiles, IU/L				*P* value^†^
	Q1 (< 79)	Q2 (79- < 96)	Q3 (96- < 116)	Q4 (≥ 116)	
N	3486	3623	3577	3707	
Antihypertensive drugs					
Calcium channel blockers	2804 (80.4)	2954 (81.5)	2958 (82.7)	3110 (83.9)	< 0.001
Diuretics	2011 (57.7)	2028 (56.0)	1901 (53.1)	1750 (47.2)	< 0.001
Lipid-lowering drugs	5 (0.1)	3 (0.1)	6 (0.2)	5 (0.1)	0.790
Antiplatelet drugs	33 (0.9)	35 (1.0)	26 (0.7)	17 (0.5)	0.045

^*^Regular concomitant medication usage was defined as 180 or more cumulative days of taking the drug of interest

^†^Difference between any 2 groups

### Association between baseline serum ALP and study outcomes

During median follow-up of 4.5 years (IQR, 4.2–4.7 years), 1549 (10.8%) participants developed new-onset diabetes. In our current study, a total of 1549 participants developed diabetes. Of these, 156 were those with physician-diagnosed diabetes, 41 reported with the use of glucose-lowering drugs during follow-up, and 1448 had a new-onset FG ≥ 7.0 mmol/L at the exit visit. Some of the patients met at least two of the above three criteria.

**Fig. 2:**
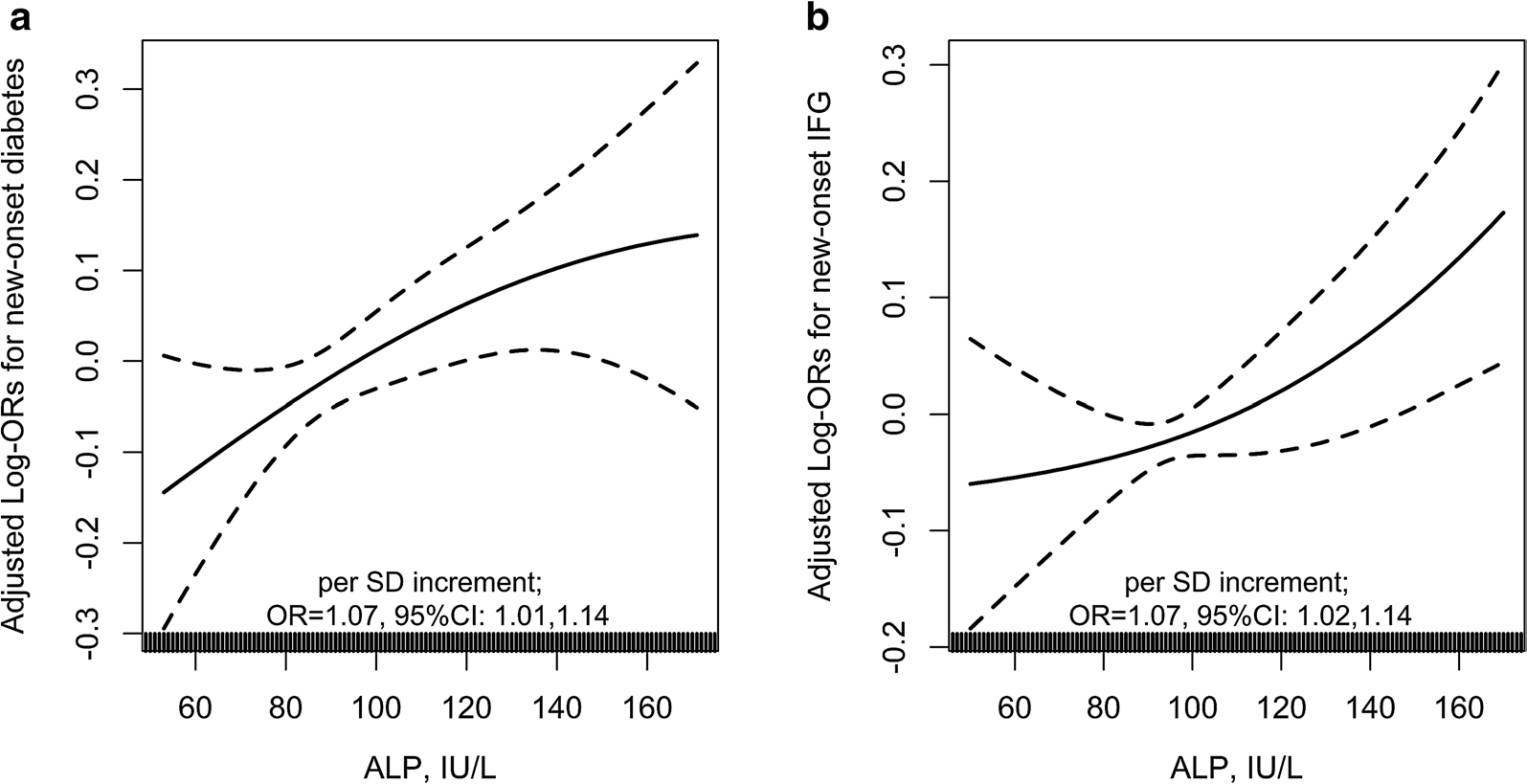
The association between baseline serum alkaline phosphatase (ALP) and new-onset diabetes **a** and new-onset impaired fasting glucose (IFG) **b** in hypertensive adults. Adjusted for age, sex, study center, treatment group, body mass index (BMI), smoking, alcohol drinking, family history of diabetes, SBP, fasting glucose (FG), total cholesterol (TC), triglycerides (TG), eGFR, folate, total homocysteine and the use of antihypertensive drugs at baseline, as well as time-averaged SBP during the treatment period. Subjects with baseline FG < 6.1 mmol/L and without new-onset diabetes during follow-up were included in the analysis for new-onset IFG

**Fig. 3:**
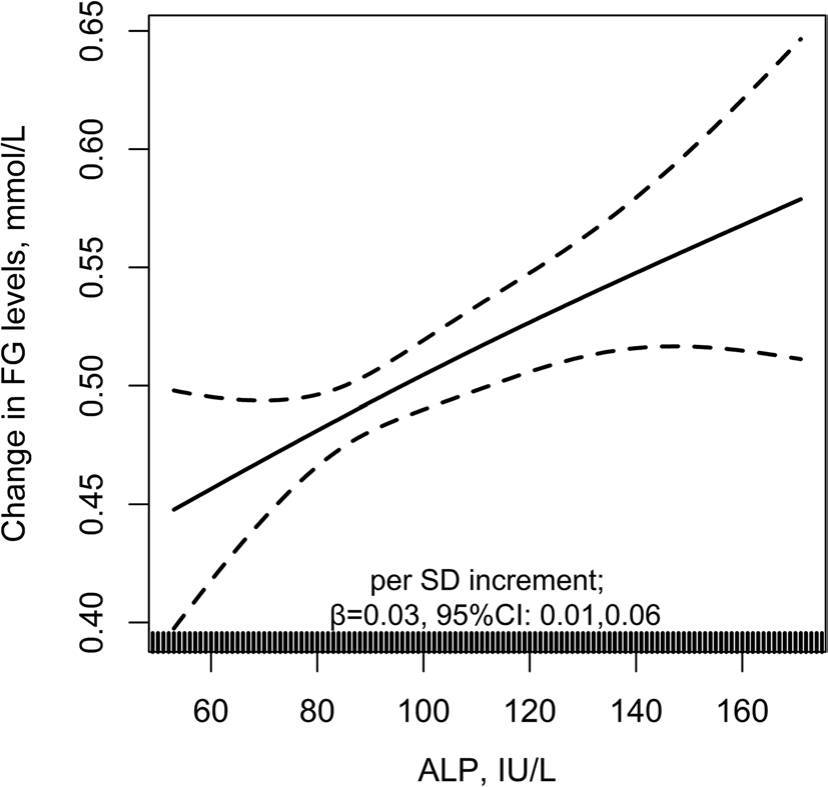
The association between baseline serum alkaline phosphatase (ALP) and change in FG levels. Adjusted for age, sex, study center, treatment group, body mass index (BMI), smoking, alcohol drinking, family history of diabetes, SBP, fasting glucose (FG), total cholesterol (TC), triglycerides (TG), eGFR, folate, total homocysteine and the use of antihypertensive drugs at baseline, as well as time-averaged SBP during the treatment period. The analysis was only included subjects without physician-diagnosed diabetes, or use of glucose-lowering drugs during follow-up

Overall, there was a positive relation of serum ALP and the risk of new-onset diabetes (per SD increment, adjusted OR, 1.07; 95% CI: 1.01, 1.14) (Fig. 2a), and new-onset IFG (per SD increment, adjusted OR, 1.07; 95% CI: 1.02, 1.14) (Fig. 2b). Consistently, compared with participants with serum ALP < 96 IU/L (median), significantly higher risks of new-onset diabetes (adjusted OR, 1.13; 95% CI: 1.00, 1.27) and new-onset IFG (adjusted OR, 1.13; 95% CI: 1.02, 1.27) were found in those with serum ALP ≥ 96 IU/L (Table 3). Accordingly, we also found a positive association between serum ALP and change in FG levels (FG level at exit visit minus that at baseline; per SD increment, adjusted β, 0.03 mmol/L; 95% CI: 0.01, 0.06) (Fig. 3).

**Table 3:** The association between baseline serum alkaline phosphatase (ALP) and study outcomes

ALP, IU/L	N	No. of events (%)	Crude model		Adjusted model*	
			OR (95% CI)	*P* value^‡^	OR (95% CI)	*P* value^‡^
New-onset diabetes						
Continuous, per SD (30.5 IU/L) increment	14,393	1549 (10.8)	1.07 (1.02,1.13)	0.007	1.07 (1.01,1.14)	0.027
Quartiles						
Q1 (< 79)	3486	343 (9.8)	1.00 (ref.)		1.00 (ref.)	
Q2 (79- < 96)	3623	381 (10.5)	1.08 (0.92,1.26)	0.346	1.09 (0.93,1.28)	0.291
Q3 (96- < 116)	3577	390 (10.9)	1.12 (0.96,1.31)	0.143	1.14 (0.96,1.34)	0.130
Q4 (≥ 116)	3707	435 (11.7)	1.22 (1.05,1.42)	0.010	1.24 (1.05,1.48)	0.013
*P* for trend			0.009		0.013	
Categories						
Q1-2 (< 96)	7109	724 (10.2)	1.00 (ref.)		1.00 (ref.)	
Q3-4 (≥ 96)	7284	825 (11.3)	1.13 (1.01,1.25)	0.027	1.13 (1.00,1.27)	0.046
New-onset IFG^†^						
Continuous, per SD (30.8 IU/L) increment	11,062	1876 (17.0)	1.04 (0.99,1.09)	0.105	1.07 (1.02,1.14)	0.012
Quartiles						
Q1 (< 79)	2629	446 (17.0)	1.00 (ref.)		1.00 (ref.)	
Q2 (79- < 96)	2788	446 (16.0)	0.93 (0.81,1.08)	0.337	0.94 (0.81,1.09)	0.415
Q3 (96- < 117)	2825	487 (17.2)	1.02 (0.89,1.17)	0.788	1.06 (0.91,1.23)	0.431
Q4 (≥ 117)	2820	497 (17.6)	1.05 (0.91,1.21)	0.520	1.14 (0.97,1.34)	0.101
*P* for trend			0.302		0.042	
Categories						
Q1-2 (< 96)	5417	892(16.5)	1.00 (ref.)		1.00 (ref.)	
Q3-4 (≥ 96)	5645	984 (17.4)	1.07 (0.97,1.18)	0.177	1.13 (1.02,1.27)	0.024

*ALP* serum alkaline phosphatase, *CI* confidence interval, *eGFR* estimated glomerular filtration rate, *IFG* impaired fasting glucose, *OR* odds ratio, *SD* standard deviations, *SBP* systolic blood pressure

*Adjusted for age, sex, study center, treatment group, body mass index (BMI), smoking, alcohol drinking, family history of diabetes, SBP, fasting glucose (FG), total cholesterol (TC), triglycerides (TG), eGFR, folate, total homocysteine and the use of antihypertensive drugs at baseline, as well as time-averaged SBP during the treatment period

^†^Subjects with baseline FG < 6.1 mmol/L and without new-onset diabetes during follow-up were included in the analysis

^‡^In comparison with the first quartile

**Fig. 4:**
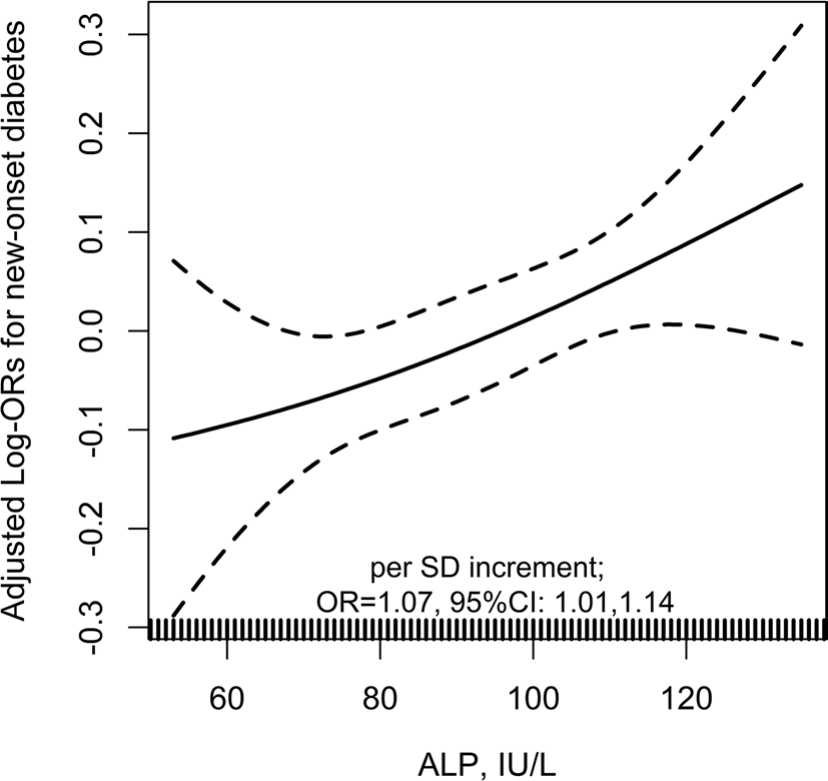
The association between baseline alkaline phosphatase (ALP) and new-onset diabetes in normal ALP levels (20–140 IU/L). Adjusted for age, sex, study center, treatment group, body mass index (BMI), smoking, alcohol drinking, family history of diabetes, SBP, fasting glucose (FG), total cholesterol (TC), triglycerides (TG), eGFR, folate, total homocysteine and the use of antihypertensive drugs at baseline, as well as time-averaged SBP during the treatment period

Similar results were also found when estimating the association between baseline ALP and new-onset diabetes with risk ratio (RR) (per SD increment; adjusted RR, 1.06; 95% CI: 1.00, 1.12) (Table 4), or in participants with a normal range of baseline serum ALP (20–140 IU/L) [26] levels (per SD increment; adjusted OR, 1.07; 95% CI: 1.01, 1.14) (Fig. 4). More importantly, further adjustment for use of calcium channel blockers, diuretics and antiplatelet drugs during the treatment period (per SD increment; adjusted OR, 1.07; 95% CI: 1.01, 1.14) (Table 5), or other liver enzymes, including GGT, ALT, AST (per SD increment; adjusted OR, 1.06; 95% CI: 1.00, 1.13) (Table 6) did not substantially change the results.

**Table 4:** Estimating the association between baseline serum alkaline phosphatase (ALP) and new-onset diabetes evaluated with risk ratio (RR)

ALP, IU/L	N	No. of events (%)	Crude model		Adjusted model*	
			RR (95% CI)	*P* value^†^	RR (95% CI)	*P* value^†^
New-onset diabetes						
Continuous, per SD (30.5 IU/L) increment	14,393	1549 (10.8)	1.06 (1.01,1.12)	0.011	1.06 (1.00,1.12)	0.034
Quartiles						
Q1 (< 79)	3486	343 (9.8)	1.00 (ref.)		1.00 (ref.)	
Q2 (79- < 96)	3623	381 (10.5)	1.07 (0.93,1.24)	0.372	1.09 (0.94,1.26)	0.274
Q3 (96- < 116)	3577	390 (10.9)	1.12 (0.97,1.29)	0.166	1.12 (0.96,1.30)	0.143
Q4 (≥ 116)	3707	435 (11.7)	1.19 (1.04,1.38)	0.015	1.21 (1.03,1.41)	0.018
*P* for trend			0.013		0.019	

*ALP* serum alkaline phosphatase, *CI* confidence interval, *eGFR* estimated glomerular filtration rate, *RR* risk ratio, *SD* standard deviations, *SBP* systolic blood pressure

*Adjusted for age, sex, study center, treatment group, body mass index (BMI), smoking, alcohol drinking, family history of diabetes, SBP, fasting glucose (FG), total cholesterol (TC), triglycerides (TG), eGFR, folate, total homocysteine and the use of antihypertensive drugs at baseline, as well as time-averaged SBP during the treatment period

^†^In comparison with the first quartile

**Table 5:** The association between baseline serum alkaline phosphatase (ALP) and new-onset diabetes, with further adjustment for the use of calcium channel blockers, diuretics and antiplatelet drugs during the treatment period

ALP, IU/L	N	No. of events (%)	Crude model		Adjusted model*	
			OR (95% CI)	*P* value^†^	OR (95% CI)	*P* value^†^
Continuous, per SD increment	14,393	1549 (10.8)	1.07 (1.02,1.13)	0.007	1.07 (1.01,1.14)	0.026
Quartiles						
Q1 (< 79)	3486	343 (9.8)	1.00 (ref.)		1.00 (ref.)	
Q2 (79- < 96)	3623	381 (10.5)	1.08 (0.92,1.26)	0.346	1.09 (0.93,1.28)	0.290
Q3 (96- < 116)	3577	390 (10.9)	1.12 (0.96,1.31)	0.143	1.14 (0.96,1.34)	0.127
Q4 (≥ 116)	3707	435 (11.7)	1.22 (1.05,1.42)	0.010	1.24 (1.05,1.48)	0.013
*P* for trend			0.009		0.013	

*ALP* serum alkaline phosphatase, *CI* confidence interval, *eGFR* estimated glomerular filtration rate, *OR* odds ratio, *SD* standard deviations, *SBP* systolic blood pressure

*Adjusted for age, sex, study center, treatment group, body mass index (BMI), smoking, alcohol drinking, family history of diabetes, SBP, fasting glucose (FG), total cholesterol (TC), triglycerides (TG), eGFR, folate, total homocysteine and the use of antihypertensive drugs at baseline, as well as time-averaged SBP, the use of calcium channel blockers, diuretics and antiplatelet drugs during the treatment period

^†^In comparison with the first quartile

**Table 6:** The association between baseline serum alkaline phosphatase (ALP) and new-onset diabetes, with further adjustment for AST, ALT and GGT

ALP, IU/L	N	No. of events (%)	Crude model		Adjusted model*	
			OR (95% CI)	*P* value^†^	OR (95% CI)	*P* value^†^
Continuous, per SD increment	14,393	1549 (10.8)	1.07 (1.02,1.13)	0.007	1.06 (1.00,1.13)	0.045
Quartiles						
Q1 (< 79)	3486	343 (9.8)	1.00 (ref.)		1.00 (ref.)	
Q2 (79- < 96)	3623	381 (10.5)	1.08 (0.92,1.26)	0.346	1.08 (0.92,1.28)	0.327
Q3 (96- < 116)	3577	390 (10.9)	1.12 (0.96,1.31)	0.143	1.13 (0.96,1.33)	0.148
Q4 (≥ 116)	3707	435 (11.7)	1.22 (1.05,1.42)	0.010	1.23 (1.03,1.46)	0.020
*P* for trend			0.009		0.019	

*ALP* serum alkaline phosphatase, *ALT* alanine aminotransferase, *AST* aspartate aminotransferase, *CI* confidence interval, *eGFR* estimated glomerular filtration rate, *GGT* gamma glutamyl transpeptidase, *OR* odds ratio, *SD* standard deviations, *SBP* systolic blood pressure

*Adjusted for age, sex, study center, treatment group, body mass index (BMI), smoking, alcohol drinking, family history of diabetes, SBP, fasting glucose (FG), total cholesterol (TC), triglycerides (TG), eGFR, folate, total homocysteine and the use of antihypertensive drugs, AST, ALT, GGT at baseline, as well as time-averaged SBP during the treatment period

^†^In comparison with the first quartile

### Association between change in serum ALP and new-onset diabetes

In order to further examine the association between serum ALP and new-onset diabetes, we investigated the relation of change in ALP with new-onset diabetes. We categorized the participants into four groups according to median of baseline serum ALP (96 IU/L): persistently low ALP levels (< 96 IU/L at both baseline and exit visit), persistently high ALP levels (≥ 96 IU/L at both baseline and exit visit), decreased ALP levels (≥ 96 IU/L at baseline and < 96 IU/L at exit visit), and increased ALP levels (< 96 IU/L at baseline and ≥ 96 IU/L at exit visit).

The incidence rates of new-onset diabetes in participants with persistently low ALP levels, persistently high ALP levels, decreased ALP levels, and increased ALP levels were 9.0, 12.6, 8.6 and 16.3%, respectively. Comparted with those with persistently low ALP levels, a significantly higher risk of new-onset diabetes was found in participants with persistently high ALP levels (adjusted OR, 1.57; 95% CI: 1.36, 1.81) or increased ALP levels (adjusted OR, 1.97; 95% CI: 1.61, 2.42); however, the adjusted odds ratio (95% CI) for those decreased ALP levels with was 0.96 (0.81, 1.14) (Table 7).

**Table 7:** The association between change in serum alkaline phosphatase (ALP) and new-onset diabetes

ALP, IU/L	N	No. of events (%)	Crude model		Adjusted model*	
			OR (95% CI)	*P* value^‡^	OR (95% CI)	*P* value^‡^
Persistently low levels^†^	6087	549 (9.0)	1.00 (ref.)		1.00 (ref.)	
Decreased levels	2744	237 (8.6)	0.95 (0.81,1.12)	0.559	0.96 (0.81,1.14)	0.653
Increased levels	993	162 (16.3)	1.97 (1.63,2.38)	< 0.001	1.97 (1.61,2.42)	< 0.001
Persistently high levels	4511	569 (12.6)	1.46 (1.29,1.65)	< 0.001	1.57 (1.36,1.81)	< 0.001

*ALP* serum alkaline phosphatase, *CI* confidence interval, *eGFR* estimated glomerular filtration rate, *GGT* gamma glutamyl transpeptidase, *OR* odds ratio, *SBP* systolic blood pressure

*Adjusted for age, sex, study center, treatment group, body mass index (BMI), smoking, alcohol drinking, family history of diabetes, SBP, fasting glucose (FG), total cholesterol (TC), triglycerides (TG), eGFR, folate, total homocysteine and the use of antihypertensive drugs at baseline, as well as time-averaged SBP during the treatment period

^†^We categorized the participants into four groups according to median of baseline serum ALP (96 IU/L): persistently low ALP levels (< 96 IU/L at both baseline and exit visit), persistently high ALP levels (≥ 96 IU/L at both baseline and exit visit), decreased ALP levels (≥ 96 IU/L at baseline and < 96 IU/L at exit visit), and increased ALP levels (< 96 IU/L at baseline and ≥ 96 IU/L at exit visit)

^‡^In comparison with persistently low ALP levels

### Stratified analyses

**Fig. 5:**
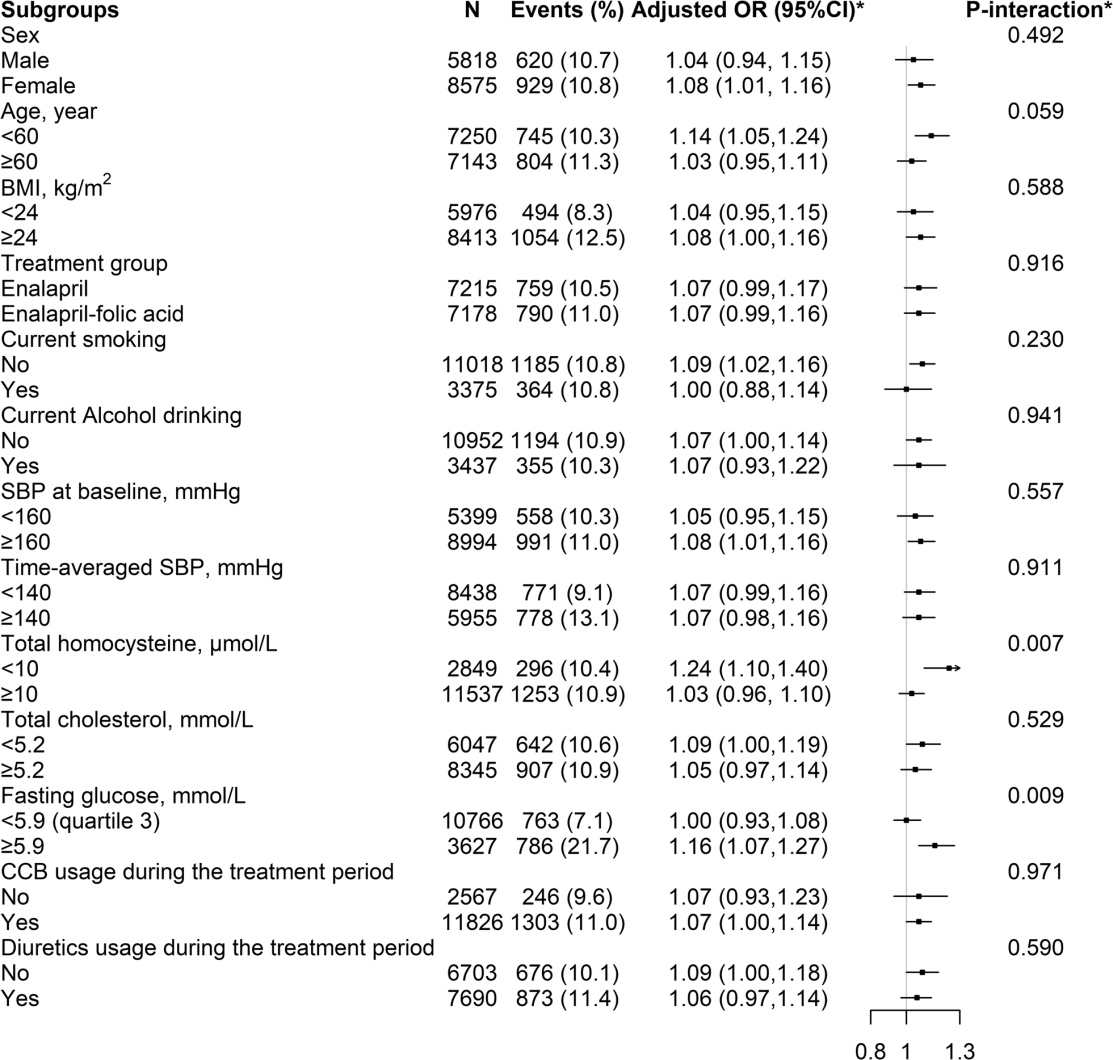
The association between baseline serum alkaline phosphatase (ALP, per SD increment) and new-onset diabetes in various groups. Adjusted for age, sex, study center, treatment group, body mass index (BMI), smoking, alcohol drinking, family history of diabetes, SBP, fasting glucose (FG), total cholesterol (TC), triglycerides (TG), eGFR, folate, total homocysteine and the use of antihypertensive drugs at baseline, as well as time-averaged SBP during the treatment period, if not be stratified

In the stratified analyses, a stronger positive association between baseline ALP with new-onset diabetes was found in participants with tHcy < 10 μmol/L (per SD increment; adjusted OR, 1.24; 95% CI: 1.10, 1.40 vs. ≥ 10 μmol/L: adjusted OR, 1.03; 95% CI: 0.96, 1.10; *P*-interaction = 0.007) and FG ≥ 5.9 mmol/L (quartile 3) (per SD increment; adjusted OR, 1.16; 95% CI: 1.07, 1.27 vs. < 5.9 mmol/L: adjusted OR, 1.00; 95% CI: 0.93, 1.08; *P*-interaction = 0.009) (Fig. 5).

However, other variables, including sex (male vs. female), age (< 60 vs. ≥ 60 years), BMI (< 24 vs. ≥ 24 kg/m^2^), treatment group (enalapril vs. enalapril-folic acid), current smoking (no vs. yes), current alcohol drinking (no vs. yes), SBP (< 160 vs. ≥ 160 mmHg), TC levels (< 5.2 vs. ≥ 5.2 mmol/L) at baseline, as well as time-averaged SBP (< 140 vs. ≥ 140 mmHg), calcium channel blockers usage (no vs. yes) and diuretics usage (no vs. yes) over the trial period, did not significantly modified the association between baseline serum ALP and new-onset diabetes (all *P*-interactions > 0.05) (Fig. 5).

## Discussion

Our study demonstrated that there was a positive association between baseline serum ALP levels and new-onset diabetes, independent of other liver aminotransferases, treated BP and other important confounders, among hypertensive patients. Moreover, our study expanded the results of previous studies by demonstrating that the positive association between baseline serum ALP levels and new-onset diabetes was more pronounced in participants with lower tHcy or higher FG levels.

### Comparisons with previous studies

Previous studies have linked serum ALP levels and the risk of diabetes, but reported controversial results. In a case–control study, Malo MS reported that high intestinal alkaline phosphatase (IAP) levels appeared to be protective against diabetes irrespective of obesity [27]. However, Nannipieri M et al. (n = 1441) [11], Nakanishi N et al. (n = 3260) [12], and Hanley AJ et al. (n = 906) [13] found that there was no significant association between ALP and incident diabetes. Moreover, a study conducted in Taiwan [14], including 132,377 non-diabetic individuals, showed that higher ALP level was significantly related to increased risk of diabetes. Of note, this study did not consider the effect of some major risk factors for diabetes, such as initial FG levels and the concomitant medications, and therefore, could not provide an accurate measurement of the association between ALP and incident diabetes. In addition, a recent mendelian randomization study demonstrated that there was a modest negative effect of genetically predicted ALP on type 2 diabetes (OR, 0.91; 95% CI: 0.86, 0.97) [28]. At the same time, another mendelian randomization study suggested that ALP was not associated with the risk of diabetes [29]. It must be pointed out that both studies [28, 29] only included European origin participants whose genetic background may be different with other population. Overall, to data, the association between ALP and incident diabetes remains uncertain. The explanations for these discrepant results might be due to differences in study population characteristics and/or sample sizes. More importantly, no previous study has comprehensively investigated the modifiers on the relation of ALP with new-onset diabetes.

### Study strengths and possible mechanisms

Our study provided a rare opportunity to evaluate the temporal and dose–response relation of serum ALP with new-onset diabetes in hypertension adults, with a comprehensive adjustment and stratified analysis for almost all the pertinent clinical information and laboratory measurements. This is the first study of this kind in a hypertensive population. Our study has made some new contributions to the field. First, we demonstrated that higher serum ALP associated with increased new-onset diabetes in hypertensive patients, independent of other liver enzymes, treated BP and traditional or suspected risk factors. Our study findings are biological plausible based on available literature, although the potential mechanisms by which serum ALP increases diabetes risk remains to be delineated. (1) ALP was reported to contribute to vascular calcification [30], which linked to insulin resistance, subsequently leading to the development of diabetes [31]. Animal experiments showed that ALP upregulation was demonstrated in the vascular wall of diabetic rat and mouse models of vascular calcification [32]. (2) Higher serum ALP was associated with increased risk of endothelial dysfunction, a process related to insulin resistance, an initial process to diabetes [33]. This was explained that ALP could reduce nitric oxide (NO) bioavailability by inhibiting tyrosine kinase activity into endothelial cells [34], leading to the consequent impairment of endothelial NO synthase function [35]. (3) Higher serum ALP levels had been reported to be associated with increased inflammation status in CKD patients or general population [36, 37]. Notably, both endothelial dysfunction [38, 39] and chronic inflammation [40, 41] has been considered as the early events in the development of the diabetes. Taken together, the aforementioned biological functions of ALP may be in part underlying our observed positive association between ALP and incident diabetes. However, more mechanistic studies are still needed.

Second, our results showed that tHcy and FG levels significantly modified the association between serum ALP and the risk of new-onset diabetes. A stronger association was found in those with lower tHcy (< 10 μmol/L) or higher FG (≥ 5.9 mmol/L) levels at baseline. The higher FG levels may partially represent the abnormal glucose metabolic state, due to the impairment of pancreatic alpha and beta cell function and the induced impaired insulin secretion [42, 43]. This population usually had a significantly increased risk of diabetes [44]. Since higher ALP was mainly associated with insulin resistance, our results suggested that increased ALP and higher FG levels may synergistically increase the risk of incident diabetes. On the other hand, it had been reported that elevated tHcy could also promote the calcification of vessels [45], and was related to endothelial dysfunction, inflammation and oxidative stress [15, 46, 47]. It seemed that elevated tHcy and ALP levels may share some common pathway in the development of diabetes. As such, the detrimental effects of higher tHcy levels may attenuate the positive relation of serum ALP levels with the risk of diabetes. Our studies suggested that the combination of optimal ALP, tHcy and FG levels may be a better strategy for the primary prevention of diabetes in hypertensive adults. However, further studies are warranted to verify this hypothesis and further examine the underlying mechanisms.

## Limitations

Our study has some limitations. First, this is a post-hoc analysis. Although our current study adjusted for a broad array of covariates in the regression models, the possibility of residual confounding cannot be excluded. Second, we did not measure glycosylated hemoglobin A1c and insulin or perform glucose tolerance tests. However, our definition of diabetes was similar to that of previous studies [48, 49]. In addition, the FG levels were assessed only at baseline and the exit visit. More frequent assays of FG levels would allow for a more accurate assessment of its progression over time. Third, in the current study, we collected total serum ALP rather than ALP isozymes. In fact, a previous case–control study had suggested that IAP deficiency was associated with type 2 diabetes mellitus [27]. However, we did not have enough blood sample for the further IAP measurements. Therefore, we could not examine the association between different ALP isozymes and new-onset diabetes. Finally, we have not available data on some diseases associated with increased serum ALP, such as Paget disease, rickets, hyperparathyroidism, osteomalacia, etc.

## Conclusions

In summary, higher serum ALP was significantly associated with increased risk of new-onset diabetes among hypertensive patients, especially in those with lower tHcy or higher FG levels. If further confirmed, our findings support the strategy to identify and modulate diabetes risk in hypertensive patients by measuring and optimizing individual serum ALP levels.

## Data Availability

The data and study materials that support the findings of this study will be available from the corresponding authors (pharmaqin@126.com) upon request, after the request is submitted and formally reviewed and approved by the Ethics Committee of the Institute of Biomedicine, Anhui Medical University.

## Abbreviations

ALP: Alkaline phosphatase
BMI: Body mass index
BP: Blood pressure
CCB: Calcium channel blockers
CVD: Cardiovascular disease
CSPPT: China Stroke Primary Prevention Trial
DBP: Diastolic blood pressure
eGFR: Estimated glomerular filtration rate
FG: Fasting glucose
HDL-C: High-density lipoprotein
IFG: Impaired fasting glucose
SBP: Systolic blood pressure
